# An ensemble-based deep learning model for detection of mutation causing cutaneous melanoma

**DOI:** 10.1038/s41598-023-49075-4

**Published:** 2023-12-14

**Authors:** Asghar Ali Shah, Ayesha Sher Ali Shaker, Sohail Jabbar, Qaisar Abbas, Talal Saad Al-Balawi, M. Emre Celebi

**Affiliations:** 1 Department of Computer Science, Bahria University, Islamabad, Pakistan; 2 Department of Computer Science, Bahria University, Lahore, Pakistan; 3 College of Computer and Information Sciences, Imam Mohammad Ibn Saud Islamic University (IMSIU), 11432 Riyadh, Saudi Arabia; 4 Department of Computer Science and Engineering, University of Central Arkansas, 201 Donaghey Ave., Conway, AR 72035 USA

**Keywords:** Cancer, Computational biology and bioinformatics, Genetics, Diseases, Health care, Mathematics and computing

## Abstract

When the mutation affects the melanocytes of the body, a condition called melanoma results which is one of the deadliest skin cancers. Early detection of cutaneous melanoma is vital for raising the chances of survival. Melanoma can be due to inherited defective genes or due to environmental factors such as excessive sun exposure. The accuracy of the state-of-the-art computer-aided diagnosis systems is unsatisfactory. Moreover, the major drawback of medical imaging is the shortage of labeled data. Generalized classifiers are required to diagnose melanoma to avoid overfitting the dataset. To address these issues, blending ensemble-based deep learning (BEDLM-CMS) model is proposed to detect mutation of cutaneous melanoma by integrating long short-term memory (LSTM), Bi-directional LSTM (BLSTM) and gated recurrent unit (GRU) architectures. The dataset used in the proposed study contains 2608 human samples and 6778 mutations in total along with 75 types of genes. The most prominent genes that function as biomarkers for early diagnosis and prognosis are utilized. Multiple extraction techniques are used in this study to extract the most-prominent features. Afterwards, we applied different DL models optimized through grid search technique to diagnose melanoma. The validity of the results is confirmed using several techniques, including tenfold cross validation (10-FCVT), independent set (IST), and self-consistency (SCT). For validation of the results multiple metrics are used which include accuracy, specificity, sensitivity, and Matthews’s correlation coefficient. BEDLM gives the highest accuracy of 97% in the independent set test whereas in self-consistency test and tenfold cross validation test it gives 94% and 93% accuracy, respectively. Accuracy of in self-consistency test, independent set test, and tenfold cross validation test is LSTM (96%, 94%, 92%), GRU (93%, 94%, 91%), and BLSTM (99%, 98%, 93%), respectively. The findings demonstrate that the proposed BEDLM-CMS can be used effectively applied for early diagnosis and treatment efficacy evaluation of cutaneous melanoma.

## Introduction

Cutaneous melanoma is a skin cancer that begins in the pigments-producing cells of the skin^1,2^. The 5-year survival rate for all people with melanoma of the skin is 93%, starting from the time of the initial diagnosis. The thickness of the primary melanoma, whether the lymph nodes are affected, and whether cancer has migrated to other locations are all factors that affect overall survival at 5 years^3^. In as little as six weeks, melanoma can spread rapidly and pose a life-threatening threat^3^. Melanoma starts to spread and advances in the stage if untreated. Superficial Spreading Melanomas (SSM), Nodular Melanoma, Lentigo malign, Acral-lentiginous melanomas, Ulcerated and/or regressive melanomas and Melanomas in special anatomical regions are a few types of cutaneous melanomas^1^. The major factor causing cutaneous melanoma is exposure to UV (ultra-violet rays) through sun^4,5^. The sequence of genes defines the structure and function of the formed organs. Any disruption or error in this specific sequence results in a mutation which, in turn, causes many abnormalities in the body. In skin cells, ultraviolet (UV) radiation can harm DNA. Sometimes the damage might affect certain genes that control cell division and growth. The affected cells may transform into cancer cells if these genes cease functioning properly^6^. However, it can be cured by performing wide local excision (WLE) if it is diagnosed and detected at initial stages. Even for professional dermatologists, identifying melanoma from skin lesions using techniques such as visual inspection, clinical screening, dermoscopic analysis, biopsy, and histological investigation can be difficult and imprecise^7–9^. Our genes, which control how our cells function, are made up of DNA, a molecule that is present in all of our cells. We resemble our parents since they are the source of our genes. But DNA affects more than just how we look. While our cells divide, develop, and die, certain genes continue to function. Oncogenes are genes that help cells grow, proliferate, and survive^10^. Tumor suppressor genes are those that regulate cell development, fix DNA errors, or induce cells to die at the appropriate moment. Cancer is produced by DNA mutations (or other sorts of alterations) that keep oncogenes active or switch tumor suppressor genes off. These sorts of gene alterations can cause cells to proliferate uncontrollably^11^. Typically, changes in multiple distinct genes are required for a cell to become a cancer cell. Most gene changes that cause melanoma to happen during a person's lifetime and are not passed on to their children. People rarely get changes in their DNA from their parents that make it clear that they are more likely to get melanoma.

**Figure 1:**
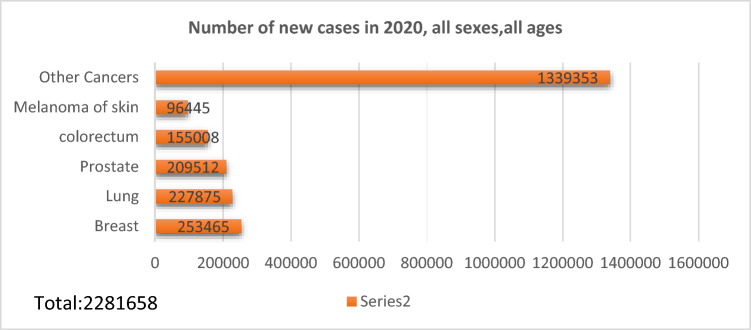
A visual example of number of new cancer cases reported for year of 2020^12^.

Skin lesions exhibit a diverse range of sizes and shapes, making their identification and classification complex. The borders of these lesions often lack distinct clarity, making it difficult to precisely delineate their edges. Moreover, when contrasted with the surrounding skin, these lesions might not exhibit a stark differentiation. Additionally, various noise elements, such as skin hair, lubricants, air, and bubbles, can further complicate the visual interpretation of skin lesions. These factors collectively contribute to the complexity of analyzing and diagnosing skin lesions, requiring advanced imaging techniques and algorithms to mitigate these challenges effectively. So, it is important to develop a reliable way to diagnose melanoma cancer. The approach suggested in this study will make it easier to diagnose melanoma early, which can make treatment easier and lower the death rate from the disease. Melanoma has the greatest fatality rate of any skin cancer, with around 10,000 cases and 12,390 fatalities recorded in the United States of America each year. New cases reported in the year of 2020 of melanoma of the skin are shown in the Fig. 1 below^12^. The most important predictor of survival in melanoma and other skin tumors is early identification^13^. Accurate skin cancer diagnosis necessitates sufficient experience and appropriate equipment. “The accuracy of melanoma diagnosis for skilled dermatologists with naked-eye inspection is less than the dermatoscopy, which is a magnifying lens with either liquid emulsion or cross-polarization filters to remove the surface reflection of skin^14,15^”. However, there is a global scarcity of skilled dermatologists^16^. Diagnosis of any disease related to skin is normally based on visual inspection and because of this different machine learning algorithms are used to develop models for detecting melanoma of skin^17^.

## Literature review

Large-scale studies on DNA sequencing have identified that driver mutations cause cancer, which could be treated at early stages. The investigation of these mutations has also yielded important insights into the biology of cutaneous melanoma of skin, which will drive future research and new treatment targets. The sequence of DNA is very important it includes reading and extracting its strands. In a study Rishitha state comparison of different deep learning and machine learning algorithms for DNA sequencing is made. dataset of sequences was used, which was 80% used for training and 20% for testing for machine learning algorithms whereas for deep learning algorithms, 75% was used for training and 25% for testing. Five algorithms from deep learning and machine learning which were CNN, Naïve Bayes, decision tree, Random Forest, and transform learning and showed the accuracy of 91.45%, 98%, 82.60%, 91.80%, 94.57%^18^. Liu in a study^19^ developed a prediction system called CanSavPre which distinguish single amino acid variation whether it was cancerous or not. Two prediction systems were developed CanSavPrew and CanSavPrewm using machine learning. Features were extracted by genetic algorithm. CanSavPrew accuracy was 79.83% with Mcc 0.45 and F1 score 0.54 wheras CanSavPrewm gave accuracy 89.73%, Mcc 0.74 and F1 score 0.81. in study^20^ genome deep learning method of deep neural network was developed as well as specific, mixture and total specific models with accuracy of 97.47%, 70.08%, and 94.70%, respectively to identification of cancer.in the study 14 models were developed. Mixture model and specific model have ratio for training and testing 80%, 20% respectively whereas total specific model randomly chose cancer samples and healthy samples of 80%. The accuracy, sensitivity, and specificity of GDL model of DNN was 97%, 98%, 97% respectively. To identify the mutation causing cancer is very difficult task and mutation recurrence is reliable indicator of their significance. Certain mutations are more likely to occur than others. Cancer driver genes often exhibit mutually exclusion of mutations, and they operate with very complex networks. The study^21^ proposed a machine learning method which investigate the functionality of mutually exclusive genes in network derived from associations of mutations, interactions of gene to gene and clustering. The method also identifies variant frequencies of driver genes and cancer related pathways which were studied less by genes whose frequencies were less. The method gave insights into cancer related mutations and its pathways to improve the understanding of the disease. The comparison of driver genes by LSFS algorithm and ShiBench, Rule, CTAT, HCD, CGC and CGCpointMut used as benchmarks in the study. The precision, recall and F-meature of ShiBench-LSFS was 0.385, 0.383, 0.384, Rule-LSFS was 0.215, 0.383, 0.275, CTAT-LSFS was 0.29,0.373,0.329, HCD-LSFS was 0.315,0.425,0.362, CGC-LSFS was 0.89, 0.50,0.640 and CGCpointMut-LSFS was 0.21,0.375,0.269 respectively.

Mutation detection would not be accurate if some sequences were lost by traditional alignment algorithms. In another study^22^ proposed feedback fast learning neural network position index algorithm for mutation detection. ACGT position index relationship in sequences of DNA. SNP and InDel mutations were studied. To analyze the linear relationship in two or more positions feedback fast learning neural network algorithms was used. Position index showed good response instead of Gatk, Vanscan2, Freebye, Bcftools for mutation detection. Position index showed good result of 84% in Exon region matching rate than the other algorithms. Location based index method showed good performance in detecting SNP and InDel. Quang in his study^23^ proposed DanQ a hybrid convolutional and bi-directional long short-term memory recurrent deep neural network model for predicting from sequences non-coding function de novo. This model learns convolution kernels and then makes them into motifs. Comparison was made between a pure CNN model which is DeepSEA and DanQ, where DanQ showed the best performance. ROC AUC of DanQ model was 94.1% whereas PR AUC of DanQ model was 97.6%. Another study was on virus mutation prediction in RNA sequences in which the author used rough set gene evolution (RSGE) and NN (neural network) techniques. The purposed techniques were trained on two different countries (Korea and China) dataset. The technique predicts nucleotides in the next generation, showing 75% accuracy whereas neural network prediction was not as good as the proposed technique. The study also analyzed the relation between nucleotides in RNA and their effect on chaining the genotype of other nucleotides^24^. In^25^ the researcher develops the new DL technique, which was DeepBind, the technique predicted the specifications of sequences of DNA and RNA binding proteins. The proposed DeepBind technique used set of sequences to compute the binding score of each sequence. Proposed technique outperforms the other methods or techniques. The technique was trained on in vitro data and tested on in vivo data. The application of DeepGPUnd to microarray and sequencing data, as well as the ability of the model to learn from millions of sequences through parallel implementation on a graphics processing unit was also discussed in the study. In another research^26^ a hybrid approach is used for detecting melanoma of skin, in this study a CNN and two other (KNN, AVM) DL approaches are used firstly individually and then combined, the combined method showed highest accuracy of 88.4% among them.

In^27^ the author of the research showed the comparison of different ML algorithms in skin cancer detection. This study showed that inception_ResNet v2 gives highest accuracy of 89.55% with 91.67% recall whereas accuracy and recall of random forest is 86.36%,95%, VGG-16 is 86.21%, 87%, ResNet-50 is 79.55%,81.67%, inception v3 is 83%,82% respectively.

Different research and their proposed system accuracy are shown in the following Table 1.

**Table 1:** Comparison of the state-of-the-art works.

Authors (citation)	Methods	Results	Drawbacks
Rishitha^18^	Decision tree, Random Forest, Naive Bayes, CNN, and transform learning	82.60%, 91.80%, 98%, 91.45%, 94.57%	The purposed transformation learning system showed 94.57% but naïve bayes showed higher accuracy than the purposed system
Liu^19^	CanSavPre_w_ and CanSavPre_wm_ a web-based tool	79.83%,89.73%	performance and accuracy should increase
Sun et al.^20^	GDL, specific, mixture and total specific models	97%, 97.47%, 70.08%, 94.70%	Genomic variation factors should be added, and model should support more cancer types
Habibi^21^	LSFS algorithm, MG algorithm	Precision of ShiBench-LSFS was 0.385, Rule-LSFS was 0.215, CTAT-LSFS was 0.29, HCD-LSFS was 0.315, CGC-LSFS was 0.89, CGCpointMut-LSFS was 0.21	Very few methods were developed, and more algorithms could be used which show more precision
Zuo et al.^22^	feedback fast learning neural network position index algorithm	High mutation detection points	Other datasets need to test on this whether they also perform good or not
Quang^23^	DanQ a hybrid convolutional and bi-directional long short-term memory recurrent deep neural network model	ROC AUC was 94.1%, PR AUC was 97.6%	Further exploration needed for different sequences
Salama et al.^24^	RSGE	Accuracy was 75%	Limited input datasets may affect the accuracy negatively and computational complexity was high
Alipanahi et al.^25^	DeepBind a deep learning technique	AUC was 0.76	Computational complexity because the model requires powerful GPU for training and calibration
Daghrir^26^	Hybrid CNN and two other (KNN, AVM) DL approaches	Accuracy was 88.4%	Shortage of data (images) availability
Bistroń^27^	Comparison of inception_ResNet v2, random forest, VGG-16, ResNet-50, inception v3	Accuracy was 89.55%, 86.36%, 86.21%, 79.55%, 83%	Impact of background on images and limited database

Mostly there are research related to the images or histopathology for detecting the melanoma of the skin using ML or DL. There are many factors which can affect the results so the novelty of the proposed study is that it can detect cutaneous melanoma of the skin earlier and efficiently and the result will be more accurate. The previous work has various limits and restrictions. There is no generic and clear benchmark dataset for cutaneous melanoma of skin-based mutations and specific sequences. Evaluation approaches are not rigorous or compelling enough. The model's accuracy has considerable potential for improvement. Keeping these constraints in mind, this study compiled the most recent and generalized datasets as described in data collection. Furthermore, numerous deep learning algorithms are used to attain the highest level of accuracy and different testing techniques are used to measure its accuracy.

## Methodology

This section includes all the step-by-step process of getting material (datasets) and processing it using different machine learning algorithms to identify melanomas. In the following sections testing methods of algorithms are explained thoroughly.

### Benchmark dataset collection

The datasets are the most critical part of the research. These datasets are used for training the model, testing, and validating the results. The Dataset is composed in such a way that it contains mutated sequences as well as normal sequences. In the proposed model to obtain the dataset of normal genes sequences are taken from the asia.ensembl.org website^28^. Mutated gene information is obtained from intogen.org^29^. To extract a dataset of normal genes sequences from asia.ensembl.org and mutated genes information from intogen.org through a web scrapping application (WSA) written in python. The database of intogen.org only has information about mutated gene so an application Mutated Sequence Generator (MSG) is developed in python language for incorporation of this information in normal genes sequence which we got from asia.ensembl.org to make mutated genes sequences. These mutated gene sequences are known as driver mutations^30^. Driver mutations are such mutations that cause cancer. The dataset used in this study was collected from 2608 cancer patients, covering 75 types of genes as outlined in Table 2. The dataset includes a total of 6778 mutated gene sequences and 6450 normal gene sequences. A set of 522 features has been extracted from each gene sequence. The problem is formulated as a binary classification task, with the target column indicating "Yes" for mutated gene sequences and "No" for normal gene sequences.

**Table 2:** Symbol of genes, number of mutations and number of samples.

Symbols of gene	Mutations	Human samples	Symbols of gene	Mutations	Human samples	Symbols of gene	Mutations	Human samples
BRAF	487	484	UBR5	67	25	KRAS	20	9
NRAS	258	256	RB1	47	24	NONO	27	8
FAT3	368	129	KIT	38	24	RET	65	8
CDKN2A	102	102	PREX2	265	24	APC	80	7
PTPRB	246	91	BCL2L12	39	22	TCIRG1	15	7
NF1	131	91	KDM5A	37	21	GNAQ	24	7
TP53	103	89	CNOT9	24	21	EPHA3	108	6
ARID2	119	86	FANCD2	50	20	ERCC3	17	5
TRRAP	160	78	IKZF1	64	18	ATR	61	5
PTEN	63	74	ZFX	24	17	HRAS	13	5
LRP1B	647	61	KMT2A	93	17	KMT2C	140	4
RGPD3	109	56	CRNKL1	34	16	QKI	8	4
PPP6C	61	55	BRCA2	69	16	MLLT3	11	4
MAP2K1	53	46	RAF1	25	13	PDCD1LG2	11	4
PRKCB	88	45	NFKBIE	20	13	CUX1	55	3
SF3B1	61	42	GNA11	19	12	HLA-A	17	3
SRGAP3	80	39	COL1A1	103	12	CDK12	27	3
NBEA	170	38	CDH11	45	11	ELL	19	2
IDH1	52	38	CYP2C8	93	11	POT1	17	2
POLQ	65	37	BRD7	24	11	SLC45A3	21	2
SETD2	58	37	BCLAF1	92	11	HSP90AB1	18	2
RAC1	46	35	CDK4	20	10	PTPRD	218	2
PBRM1	51	35	PIK3CA	31	10	FAT4	484	2
MECOM	158	29	PTPRC	119	10	AFF1	46	2
DDX3X	42	28	BAP1	21	10	PTPN6	15	2

**Figure 2:**
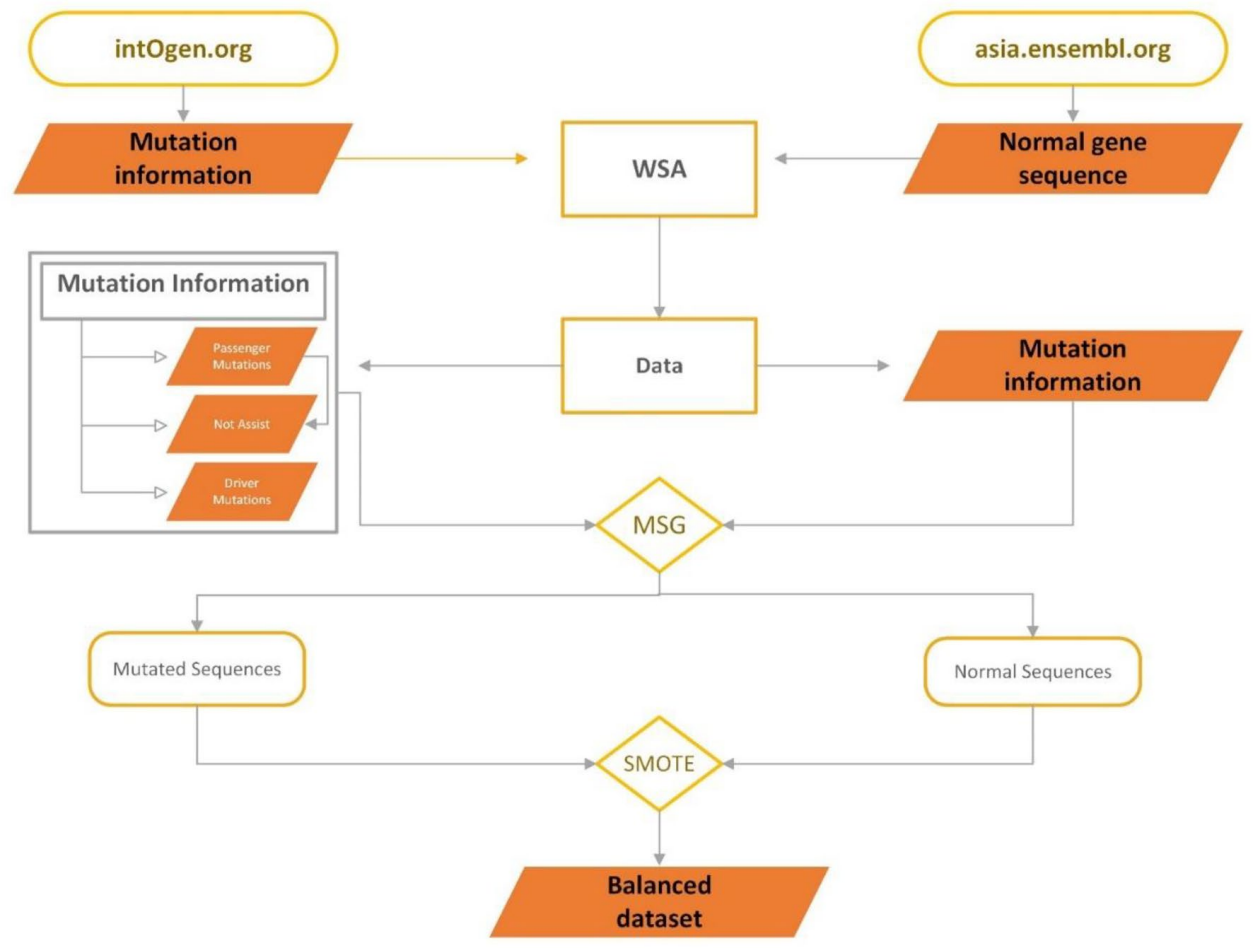
A visual example of data acquisition framework.

It is especially important to balance both datasets in the proposed framework. To balance both datasets under sampling and oversampling techniques are used. To balance the datasets The number of samples of the majority classes is reduced in the under-sampling technique whereas the number of samples increases in minority classes in the oversampling to balance datasets^31^. An oversampling technique Synthetic minority over sampling technique is used for balancing. The process of bench mark dataset collecting is also explain with the help of a Fig. 2.

For getting the most accurate results, a balanced dataset is used in the proposed study. A is the benchmark dataset in the proposed study which we get after preprocessing by CD-HIT. This dataset was processed by an ultrafast protein sequence clustering program known as CD-HIT. From each homologous cluster returned by CD-HIT, one representative was chosen., A is defined by the following Eq. (1).

1 $$A={A}^{+ }\cup { A}^{-}$$

In the equation above $\cup$ is union of both. ${A}^{+}$ is used for all mutated gene sequences that causes cancer and ${A}^{-}$ are normal gene sequences. $A$ represents the total dataset. The dataset was used in SCT (self-consistency test), IST (independent set test) and 10-FCVT (tenfold cross validation test) for training and testing purposes.

### Feature extraction

**Figure 3:**
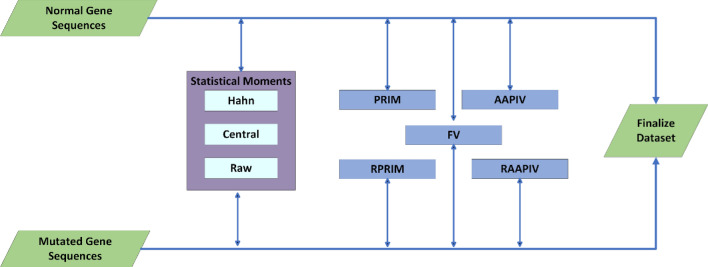
A visual example of features extraction steps by proposed method.

Feature extraction is used to improve the performance of the model. Because it reduces redundant data, so redundancy and irrelevancy are removed. Available data gives useful features by feature extraction technique^32^ as illustrated in the Fig. 3.

There are two kinds of construction models are used to present gene samples. Sequential and discrete, both modeling is mostly used to represent genes in vector formulations. The sequential model indicates the gene sequence as its nucleotide sequence which shows in the following Eq. (2).

2 $$J={N}_{1}{N}_{2}{N}_{3}{N}_{4}{N}_{5}{N}_{6}{ . . .N}_{n}$$

In the above Eq. (2) ${N}_{1}$ represent the first nucleotide in gene $J$ and ${N}_{n}$ is the last nucleotide. The total length of the sequence is $n$. In the discrete model gene sample is represented by its nucleotide composition. The gene J representation by discrete model is shown by following Eq. (3).

3 $$J={\left[ {S}_{1 } {S}_{2 } {S}_{3 } {S}_{4\dots \dots .. }{S}_{20 }\right]}^{T }$$

where ${S}_{n }\left(n=\mathrm{1,2},\mathrm{3,4},\dots 20\right)$ represent the helpful component features by extraction methods using relevant nucleotides in gene $J$. These Sequential and discrete models explained in Eqs. (2 and 3) are further used in statistical moments.

Statistical moments are used for quantitative analysis of an acquired dataset. These are applied to change data from genomic to a fixed size. Each statistical moment describes the unique information to represent the type and behavior of data. The moments size and scale of variance serve as a tool for differentiating between functionally distinct sequences. Additionally, other moments that characterize data asymmetry and mean contribute to the development of a classifier by labeled dataset. Scientists have discovered that the characteristics of genomic and proteomic sequences are affected by the content and relative location of their bases^33^. Only mathematical and computational models that are sensitive to the relative placement of component nucleotide bases within genomic sequences will be used to generate the feature vector in the future.

The gene sequences, comprising both normal and mutated variants, are acquired from a FASTA file containing gene sequences. The original dataset is structured in the gene sequences format. To enhance the dataset, we employed eight distinct feature extraction techniques: Statistical Moments (Raw, Hahn, Central), PRIM (Position Relative Incidence Matrix), RPRIM (Reverse Position Relative Incidence Matrix), AAPIV (Accumulative Absolute Position Incidence Vector), RAAPIV (Reverse Accumulative Absolute Position Incidence Vector), and FV (Frequency Vector).

To implement these techniques, we formulated and constructed the necessary algorithms. Subsequently, we developed Python code to execute these algorithms, resulting in the extraction of a comprehensive set of 522 features from each gene sequence. This systematic approach aims to capture diverse aspects of the genetic information and enhance the richness of the dataset for subsequent analyses.

Statistical parameters are computed using the Hahn moments. The Hahn moment is the key idea in pattern recognition. arithmetic mean and the variance formula. The arithmetic mean is calculated by summing up all the values in a dataset and dividing them by the number of values, while the variance measures the spread of data points around the mean^34^. Since Two-dimensional data is necessary for Hahn moments. So genomic sequences will be converted into 2D matrix ${W}^{\mathrm{^{\prime}}}$ and its size will be $X \times X$ just like following Eq. (4). It is sequential representation of the nucleotide remnant of gene covered in $W$.

4 $${W}^{{\prime} }= \left[\begin{array}{ccc}{U}_{11}& \begin{array}{cc}{U}_{12}& \cdots \end{array}& {U}_{1x}\\ {U}_{21}& \begin{array}{cc}{U}_{22}& \cdots \end{array}& {U}_{2x}\\ \vdots & \begin{array}{cc}\vdots & \vdots \end{array}& \vdots \\ {U}_{x1}& \begin{array}{cc}{U}_{x2}& \cdots \end{array}& {U}_{xx}\end{array}\right]$$

In Eq. (4) $U$ denoted as gene sequence. Values of ${W}{\prime}$ are used for computing Hahn moments. Hahn polynomial for the proposed study dataset is computed^35^ by the following Eq. (5), which is for 1D matrix of size Z.

5 $${g}_{x}^{u,v} \left(Y,Z\right)={\left(Z+M-1\right)}_{x}{\left(Z-1\right)}_{x} \times \sum_{n=0}^{x}{\left(-1\right)}^{n} \frac{{\left(-x\right)}_{n}{\left(-Y\right)}_{n}{\left(2Z+u+v-x-1\right)}_{n}}{{\left(Z+v-1\right)}_{n}{\left(Z-1\right)}_{n}} \frac{1}{n!}$$

Here u and v are all positive integers and predefined constants. The order of the moment is x and Z is the size of data. The Hahn moment calculated up to 3rd order for discrete 2D data is in following Eq. (6) as:

6 $${Q}_{gh}=\sum_{s=0}^{R-1}\sum_{t=0}^{R-1}{ \delta }_{gh}{f}_{g}^{l,m}\, \left(k,Z\right){f}_{h}^{l,m}\left(s,Z\right),t,y=\mathrm{0,1},2,\dots ,Z-1$$

“Here $g+h$ is the order of the moment, $l,m$ are predefined constants and ${\delta }_{gh}$ is an arbitrary element of matrix ${W}{\prime}$. Equations (5) and (6) are employed to determine the normalized Hahn moment of any order efficiently. The unique features of Hahn moments up to ${3}^{rd}$ order is as ${Q}_{00,}{Q}_{01,}{Q}_{10,}{Q}_{11,}{Q}_{02,}{Q}_{20,}{Q}_{12,}{Q}_{21,}{Q}_{03 }\& {Q}_{30}$^36^. In total, we have calculated 10 Hahn moments for every gene sequence up to ${3}^{rd}$ order”.

When calculating the mean, variance, and asymmetries of a probability distribution, raw moments are employed. Neither scale invariance nor location invariance applies to these raw moments. For statistics imputation, raw moment is utilized. Imputation is the process of replacing missing data values in a dataset with the best substitute values to preserve information^37,38^.

Raw moments are computed by using the values of ${W}{\prime}$. The raw moments $Z \left(j, k\right)$ of order $j+k$ are computed by the following Eq. (7).

7 $${Z}_{jk}= {\Sigma }_{m}{\Sigma }_{n}{m}^{j}{n}^{k}{W}^{{\prime} }\left(m,n\right)$$

“The origin of data is used as the reference point from which raw moments are computed, and the origin is used as the distance between the components. Above equation computed raw moments up to ${3}{rd}$ order”. Raw moments features are as ${Z}_{00}$, ${Z}_{01}$, ${Z}_{10}$, ${Z}_{11}$, ${Z}_{02}$, ${Z}_{20}$, ${Z}_{12}$, ${Z}_{21}$, ${Z}_{03} \& {Z}_{30}$. For every gene sequence 10 Raw moments are calculated up to ${3}{rd}$ order.

The centroid, also known as the geometric center or center of gravity, holds significance in both geometry and data analysis. In geometric figures, it represents the average position of all points on the shape's surface and serves as the intersection point of its medians. This point is often equated to the center of mass due to its influence on the figure's balance. In data analysis, the centroid extends its meaning to the average location of data points in a multi-dimensional space, with each dimension corresponding to a variable. Overall, the centroid stands as a pivotal notion, bridging the realms of geometry and statistics, providing insights into central tendencies and structural characteristics of shapes and datasets. A data point from which all data is dispersed equally in all directions. These directions are weighted average relationships^39–41^.

Unique features of central moment calculated up to 3^rd^ order by the following Eq. (8) with the help of centroid of data as reference point.

8 $${M}_{jk}= {\sum }_{m}{\sum }_{n}{\left(m-\overline{m }\right)}^{j}{\left(n-\overline{n }\right)}^{k}{ W}^{{\prime} }\left(m,n\right)$$

Central moments unique features up to ${3}^{rd}$ order are as ${M}_{00},{M}_{01},{M}_{10},{M}_{11},{M}_{02},{M}_{20},{M}_{12},{M}_{21},{M}_{03},\&{M}_{30}$.centroids of central moments are computed as $\overline{m }$ and $\overline{n }$ as following Eqs. (9) and (10):

9 $$\overline{m }=\frac{{Z}_{10}}{{Z}_{00}} ,$$

10 $$\overline{n }=\frac{{Z}_{01}}{{Z}_{00}} $$

Ten central moments are also calculated for every gene sequence up to 3^rd^ order. These 10 unique features of Hahn moment, 10 of unique features of Raw moment and 10 of unique features of central moment, which we got then further unified as a SFV (Super Feature Vector).

To identify the genetic characteristics and the ordered location of the nucleotides in gene sequences is very important^41,42^. “The relative position of nucleotides in any gene sequence is seen as a fundamental pattern that makes use of the physical properties of the gene sequence^43,44^. PRIM represent the gene sequence in (20 × 20) order. The relative position of all nucleotides in the given gene sequence is extracted by the following matrix in Eq. (11)”:

11 $${O}_{PRIM}= \left[\begin{array}{cccc}{O}_{1\to 1}& \begin{array}{cc}{O}_{1\to 2}& \cdots \end{array}& \begin{array}{cc}{O}_{1\to n}& \cdots \end{array}& {O}_{1\to C}\\ {O}_{2\to 1}& \begin{array}{cc}{O}_{2\to 2}& \cdots \end{array}& \begin{array}{cc}{O}_{2\to n}& \cdots \end{array}& {O}_{2\to C}\\ \vdots & \vdots & \vdots & \vdots \\ {O}_{m\to 1}& {O}_{m\to 2}& {O}_{m\to n}& {O}_{m\to C}\\ \vdots & \vdots & \vdots & \vdots \\ {O}_{C\to 1}& {O}_{C\to 2}& {O}_{C\to n}& {O}_{C\to C}\end{array}\right]$$

In the above equation ${O}_{m\to n}$ denotes the cluster of the relative positions of the $nth$ base regarding the initial occurrence of the $mth$ base. Further by using this 2D ${O}_{PRIM}$ matrix Hahn, Raw and central moments were calculated.

The process of R-PRIM and PRIM calculations is the same, however, only R-PRIM works with reverse gene sequence ordering^35^. R-PRIM computing revealed underlying patterns, allowing discrepancies between homologous sequences to be resolved. R-PRIM was also constructed as a 2D matrix of order (20 × 20) with 400 coefficients. R-PRIM matrix represents as following Eq. (12):

12 $${O}_{RPRIM}= \left[\begin{array}{cccc}{O}_{1\to 1}& \begin{array}{cc}{O}_{1\to 2}& \cdots \end{array}& \begin{array}{cc}{O}_{1\to n}& \cdots \end{array}& {O}_{1\to C}\\ {O}_{2\to 1}& \begin{array}{cc}{O}_{2\to 2}& \cdots \end{array}& \begin{array}{cc}{O}_{2\to n}& \cdots \end{array}& {O}_{2\to C}\\ \vdots & \vdots & \vdots & \vdots \\ {O}_{m\to 1}& {O}_{m\to 2}& {O}_{m\to n}& {O}_{m\to C}\\ \vdots & \vdots & \vdots & \vdots \\ {O}_{C\to 1}& {O}_{C\to 2}& {O}_{C\to n}& {O}_{C\to C}\end{array}\right]$$

Just like PRIM R-PRIM is also used for calculating Hahn, raw and central moments.

“The frequency vector is easily calculated by counting the number of times each nucleotide residue appears in the main sequence. The frequency vector's elements reflect the frequency of occurrence of the relevant nucleotide residue within the supplied sequence. As a result, the frequency vector has 20 coefficients”. The following Eq. (13) represents frequency vector as:

13 $$\sigma =\left\{{\rho }_{1},{\rho }_{2},{\rho }_{3},\dots ..,{\rho }_{n}\right\}$$

In the above equation the frequency of each nucleotide in gene sequence is $\rho$. These measurements are used to alleviate information on the position importance of nucleotide in a sequence. 20 FV (frequency vector) features are also integrated into the SFV (Super Feature Vector).

Feature extraction is a successful method for obtaining confusing patterns in gene sequences. Accumulative absolute position incidence vector (AAPIV) provides accumulative information regarding the position occurrence in gene sequences for each nucleotide base^38^. The placement of gene sequences of cutaneous melanoma is shown in Eq. (14) as:

14 $$P=\left\{{\xi }_{1},{\xi }_{2},{\xi }_{3},{\xi }_{4},\dots {\xi }_{n}\right\}$$

Here ${\xi }_{n}$ is gene sequence which have n total nucleotides, these nucleotides can be computed by using the following Eq. (15) as for any $ith$ component.

15 $${\xi }_{i}= \sum \limits_{l=0}^{n}{J}_{k}$$

In the above equation ${\xi }_{i}$ is from gene sequence ${J}_{k}$ which have n numbers of nucleotides. AAPIV was used to accommodate relative positioning information from 20 native nucleotides in a gene sequence with a length of 20 related important features. These 20 important AAPIV elements are likewise integrated into the miscellaneous SFV (Super Feature Vector).

The reverse sequencing provides a more in-depth view of the hidden patterns in the gene sequence. RAAPIV refers to the computation of AAPIV for the reverse sequencing of the gene^34^. It is written as:

16 $$R=\left\{{\xi }_{1},{\xi }_{2},{\xi }_{3},{\xi }_{4},\dots {\xi }_{20}\right\}$$

Formation of RAAPIV is mentioned in the above Eq. (16) in which 20 unique features are made. These 20 unique features are then added up into SFV.

17 $${\xi }_{i}= {\sum }_{l=0}^{n}{Reverse\left(J\right)}_{k}$$

In the above equation ${\xi }_{i}$ is element of R-AAPIV from gene sequence ${J}_{k}$ which have n number of nucleotides which is calculated by the above Eq. (17). Unique features are extracted from all the above-mentioned methods and SFV (Super Feature Vector) is created having 150-D number of features. This SFV (Super Feature Vector) is further used in prediction algorithms mentioned below.

### Prediction algorithms

In this proposed study, a deep neural network with multiple layers is employed to identify cutaneous melanoma of the skin. Deep learning plays a significant role in the recognition, detection, prognosis, diagnosis, forecasting, and detection systems related to cutaneous melanoma. The deep neural network model comprises various layers, including an input layer, an output layer, a pooling layer, a dense layer, and a dropout layer, with fully connected layers stacked on top^45^. Each layer accepts input from the preceding layer and processes the input features. These layers incorporate learning characteristics that self-educate using various learning techniques^46^.

**Figure 4:**
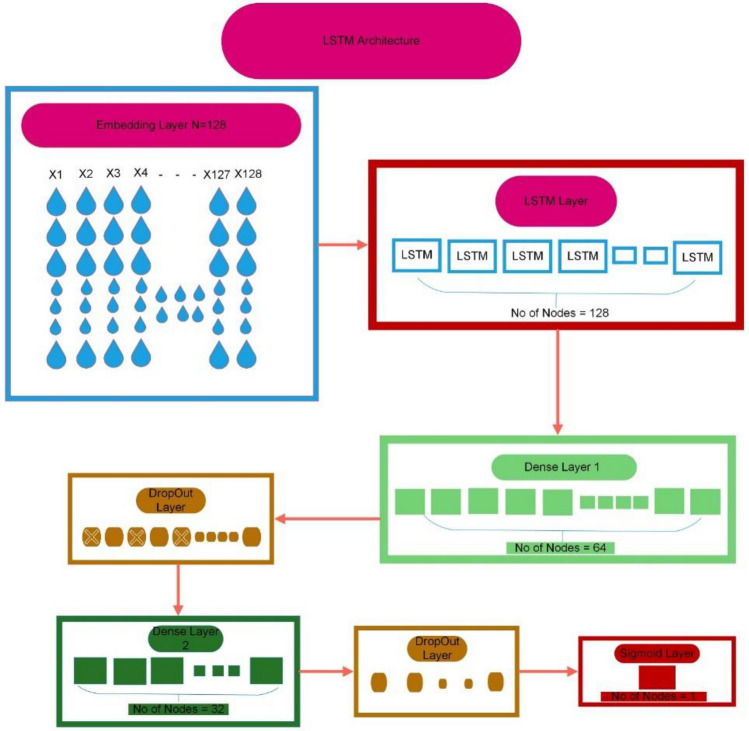
A LSTM architecture.

Within this work, three types of deep learning recurrent neural network (RNN) algorithms are utilized: Long Short-Term Memory (LSTM), Gated Recurrent Units (GRU), and Bidirectional LSTM^47^. These algorithms employ three assessment methods for the detection of cutaneous melanoma: a self-consistency test, an independent set test, and a tenfold cross-validation test. The first deep learning algorithm employed in this procedure is LSTM, chosen for its ability to address the vanishing gradient problem encountered in neural networks. The vanishing gradient problem occurs when the loss function approaches zero, making training neural networks challenging. LSTM is specifically utilized in recurrent neural networks to mitigate short-term and vanishing gradient issues by extending the network's memory capacity. It operates through a gated process involving three types of gates: input gates, forget gates, and output gates^48^. Each gate has a distinct role in regulating the flow of information from one stage to another. Consequently, unique activation functions are applied to each gate. Additionally, the suggested LSTM architecture includes an embedding layer. Figure 4 illustrates the LSTM architecture used in this proposed study.

The gate in LSTM oversees regulating information flow from one cell to another. Each gate uses a different activation function^49,50^. The following Eqs. (18,19,20,21,22,23) show the working of LSTM.

18 $${{\text{g}}}_{{\text{t}}}=\upsigma ({z}_{t}{X}^{g}+{j}_{t-1}{Y}^{g})$$

The data is entered in LSTM layer from embedding layer. In LSTM layer data is passed through LSTM gates mentioned in above equations. In our proposed model, the embedding layer serves to convert input data into a fixed-length vector of a specified size. The vocabulary size is set at 1000, and the word vectors have a length of 64. Following the embedding layer, we incorporate an LSTM layer as the second layer, which features an output layer housing 128 neurons. Additionally, two dropout layers are introduced, with 10% of neurons deactivated, effectively addressing the issue of overfitting. A dense layer with 10 neurons is included in order to add depth to the network. Stochastic Gradient Descent (SGD) is employed as the optimizer within the LSTM layer, and the sigmoid function serves as the activation function. To minimize the loss, the Sparse Categorical Cross Entropy (SCCE) function is utilized.

**Figure 5:**
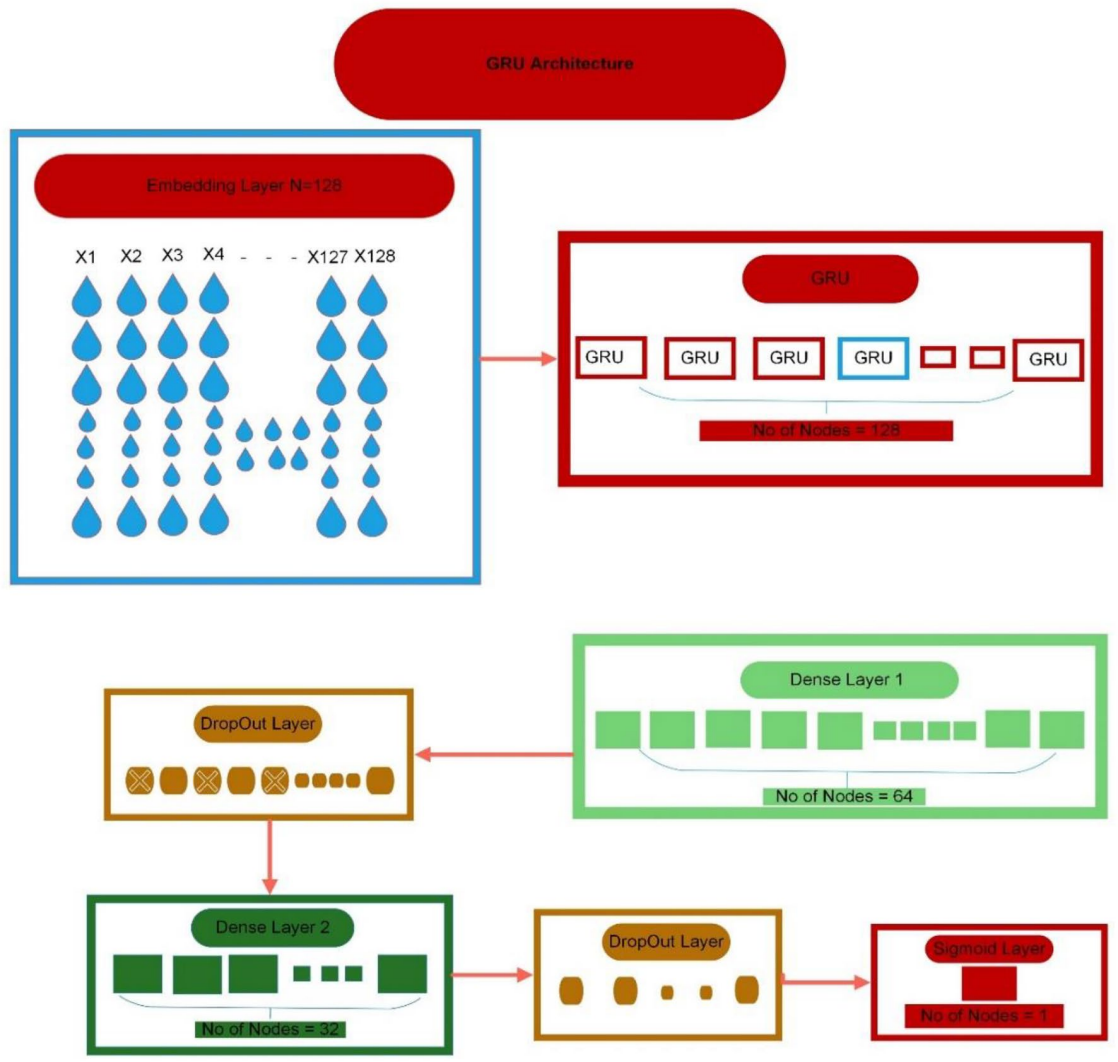
A GRU architecture.

In the context of this study, the Gated Recurrent Unit (GRU) approach is the second deep learning method implemented. GRU exhibits fewer gates compared to LSTM but performs analogous functions. Notably, due to its reduced number of gates and parameters, GRU tends to yield superior results compared to LSTM. In the cell, GRU relies on just two gates: the reset gate and the update gate. The reset gate dictates the extent to which prior information is disregarded, while the update gate influences the extent to which past information is incorporated^48^. GRU also boasts faster computational speed when compared to LSTM^51^. Figure 5 provides a visual representation of the GRU architecture.

In our proposed model, a single embedding layer is utilized to transform input data into a vector with a fixed word length of 64. Subsequently, the second layer consists of a GRU layer housing 256 neurons, accompanied by a basic RNN layer featuring 128 neurons. To avoid overfitting, two dropout layers are introduced at 30%. Towards the end, a considerable layer of 10 neurons is added. In the GRU layer, Stochastic Gradient Descent (SGD) is implemented as an optimizer. The sigmoid function is applied as an activation function. To lessen the loss suffered when training the suggested model, Sparse Categorical Cross Entropy (SCCE) is adopted. Following Eqs. (19), (20), (21), (22), (23) show the working of the GRU.

19 $${s}_{t}= \sigma \left({g}_{t}{K}^{s}+{J}_{t-1}-{Z}^{s}\right)$$

20 $${n}_{t}= \sigma \left({g}_{t}{K}^{n}+{J}_{t-1}-{Z}^{n}\right) $$

21 $${j}_{t}{\prime}=tanj \left({s}_{t}*{J}_{t-1}K+ {g}_{t}Z \right) $$

Here ${s}_{t}$ represent reset gate and ${n}_{t}$ is updating gate.

22 $${j}_{t}{\prime}=tanj \left({s}_{t}*{J}_{t-1}K+ {g}_{t}Z \right)$$

23 $${p}_{t}=\left(1-{n}_{t}\right)*{J}_{t}^{\mathrm{^{\prime}}}+{n}_{t}*{J}_{t-1})$$

**Figure 6:**
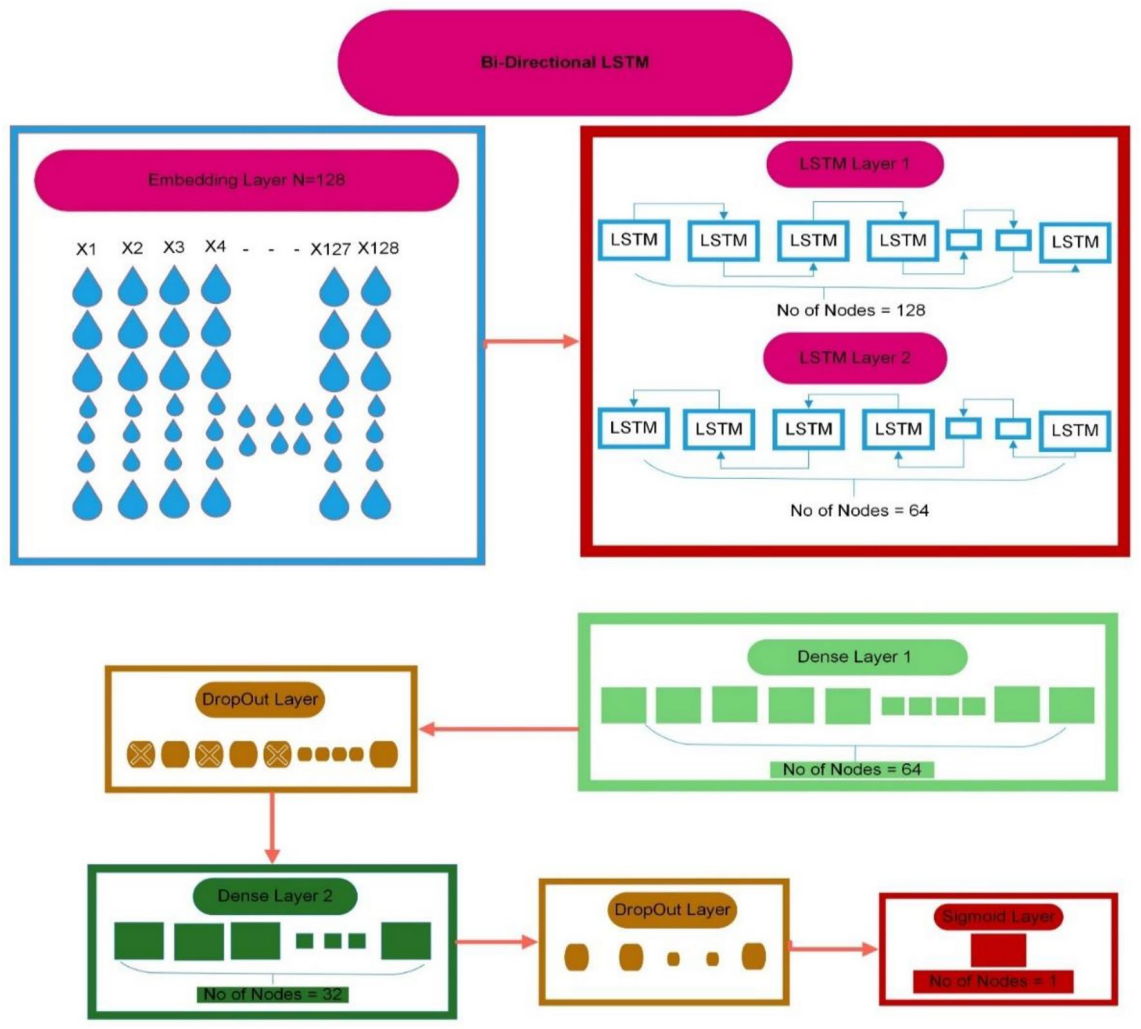
A proposed BLSTM architecture.

In the final stage of our proposed study, we utilize a bi-directional LSTM as our chosen deep learning approach. A bi-directional LSTM connects two LSTM cells, one operating in the forward direction and the other in the backward direction, ultimately yielding a single output^52^. In our suggested model, an embedding layer is employed to transform input data into fixed-length vectors, each consisting of 64 words. We incorporate two bi-directional layers, each featuring 128 neurons in the forward direction and 64 neurons in the backward direction. To prevent overfitting, three dropout layers are introduced at 30%. One dense layer with 64 neurons is employed, and one dense layer with 10 neurons is added at the end. In the GRU layer, Stochastic Gradient Descent (SGD) is utilized as an optimizer. The sigmoid function is utilized as an activation function as shown in the Fig. 6. To minimize the loss in training the suggested model, Sparse Categorical Cross Entropy (SCCE) is utilized”.

The goal of all these models is to achieve high accuracy. Unlike LSTM and GRU, Bi-directional LSTM does not require any prior information for prediction; it learns on its own by going ahead and backward, which is why the outcome of Bi-directional LSTM is superior to LSTM and GRU^53^.

**Figure 7:**
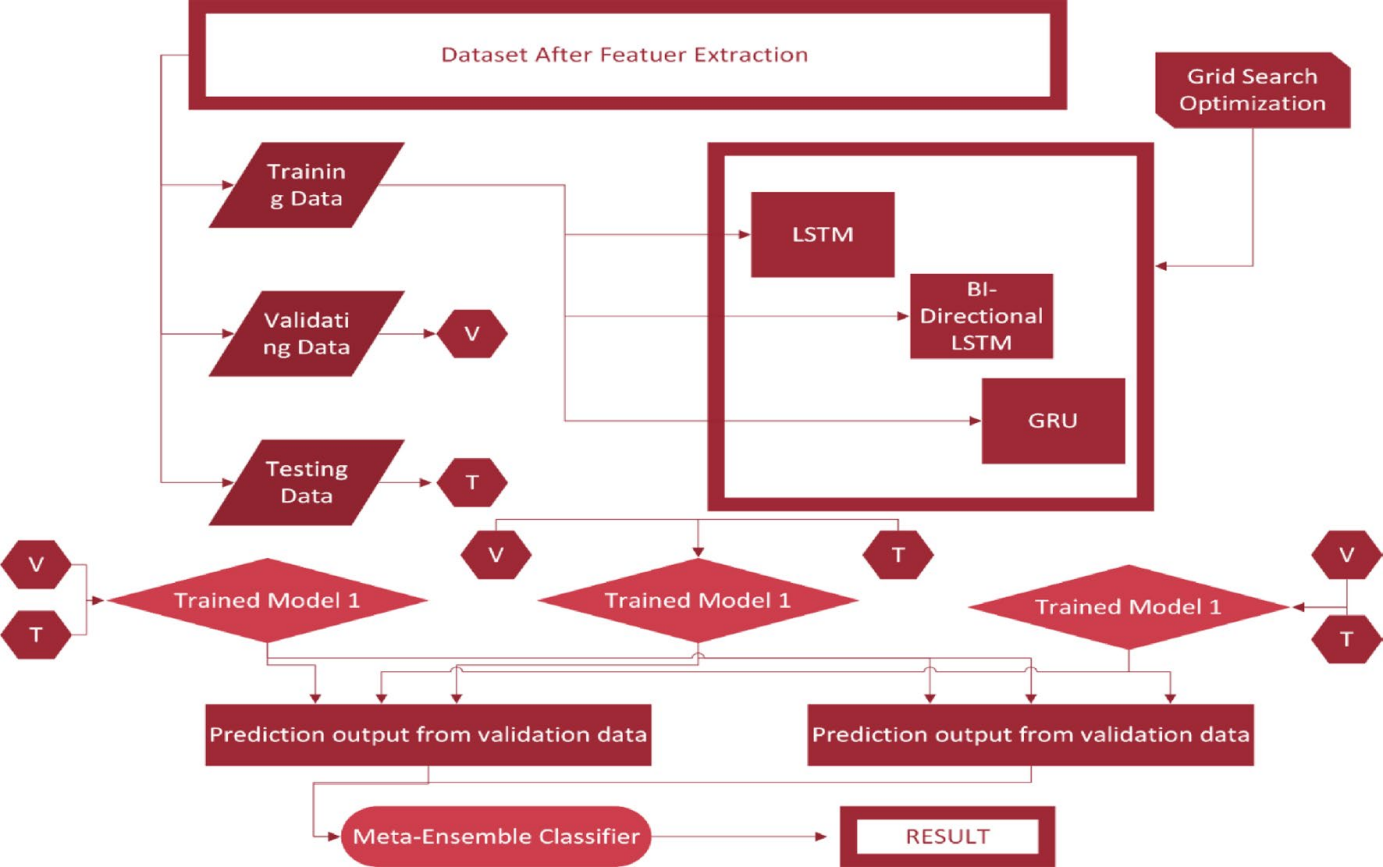
A proposed BEDLM architecture.

The BEDLM employs a divide-and-conquer strategy. It is used to increase the accuracy of a single base learner before compiling the entire model. To produce the best outcomes, many base learners are blended. Each base learner extracts various characteristics from data chunks received using the bootstrap process, creates some outcomes, and combines them. The data pieces are then sent back into the model. The model learns the patterns hidden in the datasets in this manner. BELDM is a flexible technique that outperforms simple machine learning algorithms in terms of accuracy. This is because the bootstrap approach allows for feature and row replacement strategies, and the model learns utilizing all conceivable data combinations. This also leads to the resolution of the overfitting difficulties. Bagging^54^, boosting^55^, blending, and stacking^56^ are four prominent ensemble deep learning model types. The goal of all these models is to achieve high accuracy. Blending of EDLM type is used in the proposed study^57^. BEDLM improved the performance of all the above-described deep learning models such as LSTM, GRU and Bi-directional LSTM.As shown in the following Fig. 7, dataset which we processed above are divided into three groups such as training dataset, validation dataset (denoted as V) and testing (denoted as T) dataset. The training dataset used as an input to all the DL models such as LSTM, GRU and Bi-directional LSTM. For all DL models trained models are developed which are trained model no. 1, trained model no.2, trained model no. 3. After that trained model no. 1, trained model no. 2, trained model no. 3 is tested on validation dataset and testing dataset. A result which is obtained from this model are shown in the result Table 3.

**Table 3:** Results of SCT by using LSTM, GRU, BLSTM and BEDLM.

Deep learning models	Self-consistency test (SCT)			
	ACC%	SE%	SP%	MCC
GRU	93	95	92	0.87
BLSM	99	100	97	0.97
Proposed BEDLM	94	95	93	0.88
LSTM	96	97	94	0.92

24 $$pt,j=\sum \limits_{m=1}^{M}{w}_{m}{f}_{m,j } $$

All DL models are assigned weights to make BEDLM described in the equation where ${w}_{m}\left(m=\mathrm{1,2},\mathrm{3,4},\dots .,M\right)$ is the weight and ${f}_{m,j}$ is the prediction of all DL models and $m$ is for jth observation. These testing strategies are used in 10 spans for each DL methodology which is 10 feed-forward and feed backward paths. The model determines it is ROC, specificity, sensitivity, Mathew's correlation coefficient and accuracy in each testing iteration.

## Results

The cutaneous melanoma of skin dataset is firstly preprocessed and after that processed to obtain the essential aspects of the balanced data. The retrieved data is subsequently subjected to the deep learning (DL) algorithms. The independent set test, self-consistency test, and tenfold cross-validation test is used to validate the performance of deep learning (DL) algorithms. This section explains the findings of these validation approaches.

**Figure 8:**
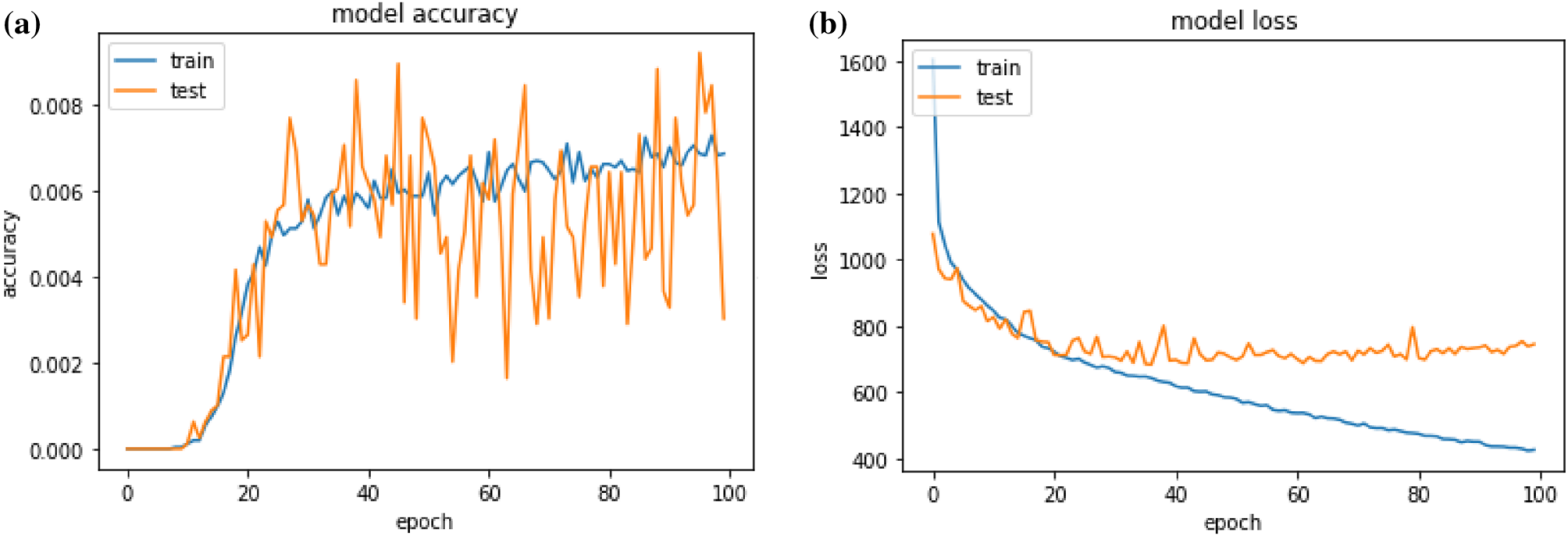
The figure shows the (**a**) train and validation accuracy, whereas the figure (**b**) represents train and validation loss graphs of the proposed architecture. BEDLM.

In the proposed deep learning model, Fig. 8 illustrates the accuracy and loss values during the training and testing phases. The experiment is conducted using a tenfold cross-validation technique. Each fold comprises measurements such as training accuracy (Acc), testing accuracy (Val Acc), training loss (Loss), and testing loss (Val Loss) for both training and testing samples.

The mathematical formulas of sensitivity, specificity, accuracy and Matthew’s correlation coefficient utilized to calculate the outcomes of the algorithms.^58^. Sensitivity which is also called recall is called true positive value is the probability of a positive value in target condition. Specificity is also called true negative values is the negative value in the target condition. Accuracy is the proportion of properly identified samples to the total number of samples. MCC (Matthew correlation coefficient) calculates the difference be-tween expected and actual values.

False negatives (FN) are examples of negative data that the system mistakenly interpreted as positive, while true positives (TP) are examples of positive data that the system correctly identified. False positives (FP) are good things that are wrongly thought to be good, while true negatives (TN) are bad things that are correctly called bad (FP). Here, the capacity to anticipate the count that properly identifies the melanoma of skin is referred to as sensitivity and the capacity to forecast the count that accurately identifies the absence of melanoma of skin is referred to as specificity^59^. All subjects with the specified condition are represented by P + FN. TN + FP are subjects who do not have the stated criteria. The total number of participants with positive findings is TP + FP, while the total number of subjects with negative results is TN + FN^57^.

### Self-consistency test (SCT)

**Figure 9:**
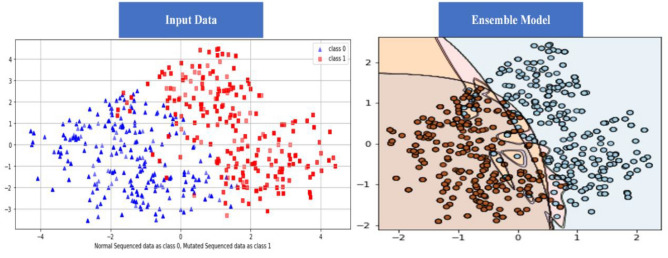
Decision Boundary of SCT in BEDLM.

**Figure 10:**
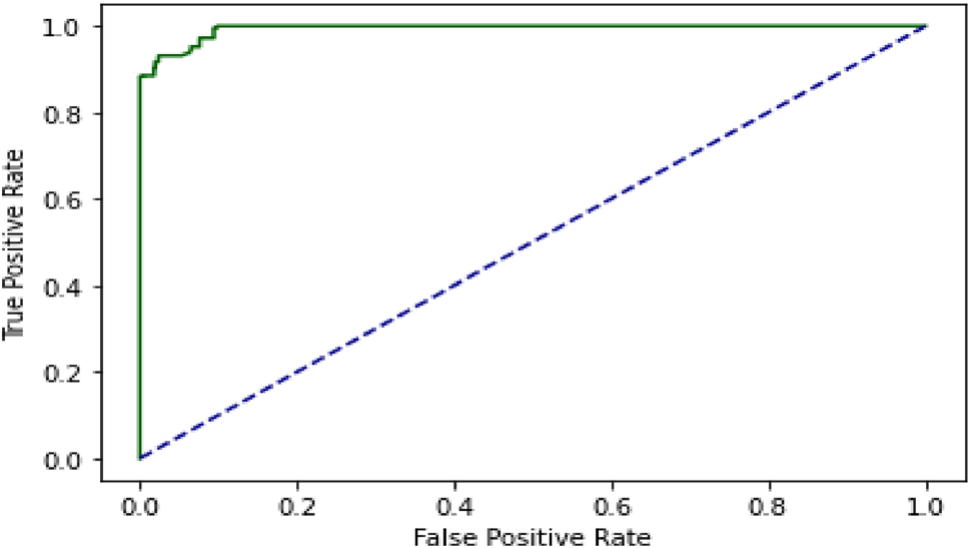
A visual example of receiver-operating** (**ROC) curve in SCT of BEDLM.

The DL algorithm is tested using the SCT approach. 100% data is utilized for training and testing in the SCT. The entire dataset is used for both training and testing in the SCT. The loss in bidirectional LSTM is quite low. LSTM, GRU, and Bidirectional LSTM, on the other hand, achieved very excellent accuracy in the SCT, as demonstrated in the result Table 3. The decision boundary of SCT is shown in the Fig. 9 as below. ROC curve (receiver operating characteristic curve) of SCT is shown in the Fig. 10 as below.

### Independent set test (IST)

**Figure 11:**
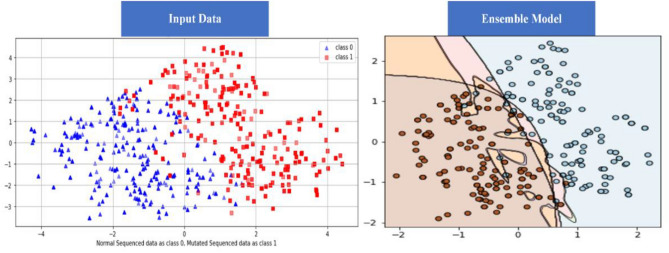
Decision Boundary of SCT in BEDLM.

**Figure 12:**
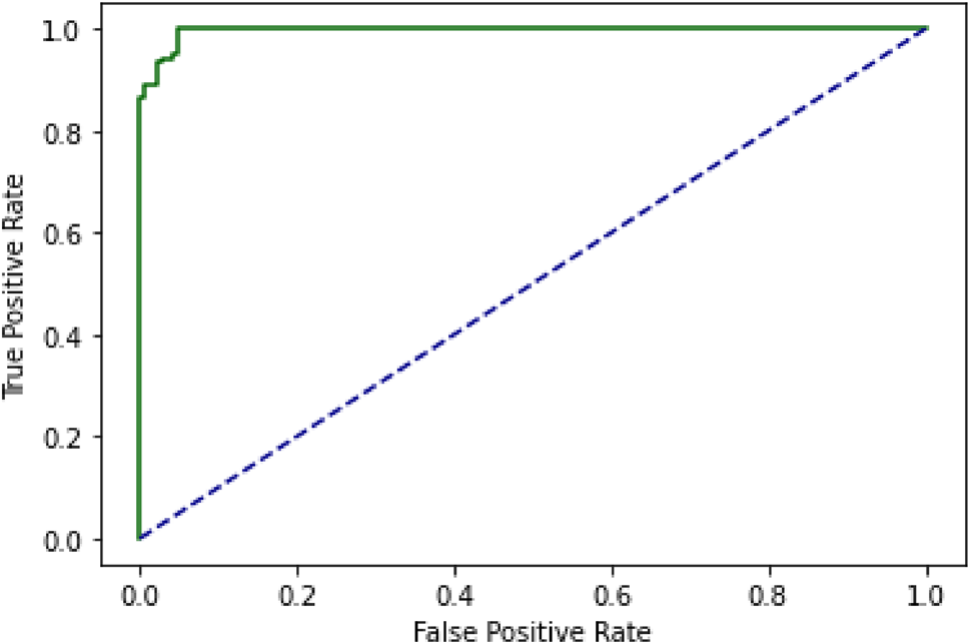
A visual example of receiver-operating** (**ROC) curve in SCT of BEDLM.

IST is the second testing technique utilized for the suggested BEDLM strategy. The values are retrieved from the misperception matrix, which is used to calculate the model's accuracy. The suggested model's IST is the primary performance measurement approach. 80% of the values in the dataset is used to train the algorithm, while 20% is utilized for testing. The decision boundary of IST is illustrated as Fig. 11 below. ROC curve (receiver operating characteristic curve) of the IST is illustrated as Fig. 12 below. The Independent set test results are shown in Table 4.

**Table 4:** Results of IST by using LSTM, GRU, BLSTM and BEDLM.

Deep learning models	Independent set test (IST)			
	ACC%	SE%	SP%	MCC
GRU	94	96	92	0.89
BLSM	94	96	91	0.87
Proposed BEDLM	98	100	95	0.95
LSTM	97	99	95	0.94

### Tenfold cross validation test (10-FCVT)

**Figure 13:**
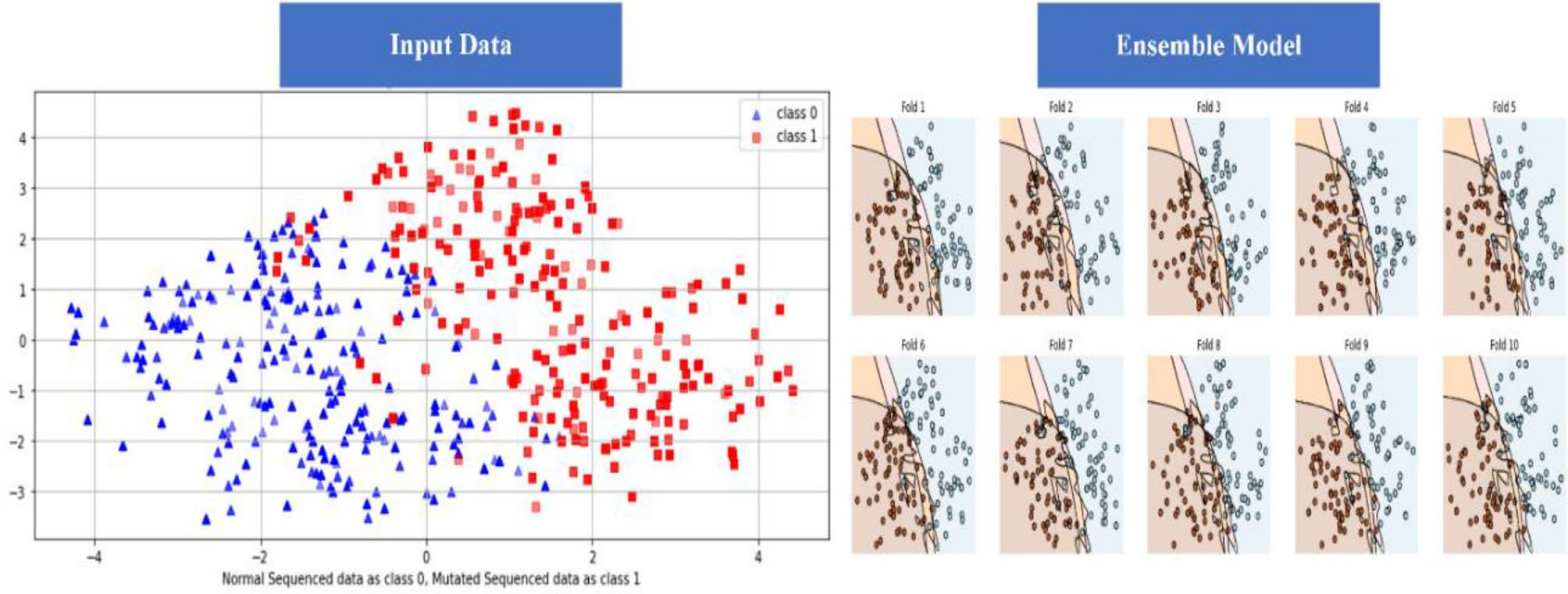
Decision Boundary of SCT in BEDLM.

**Figure 14:**
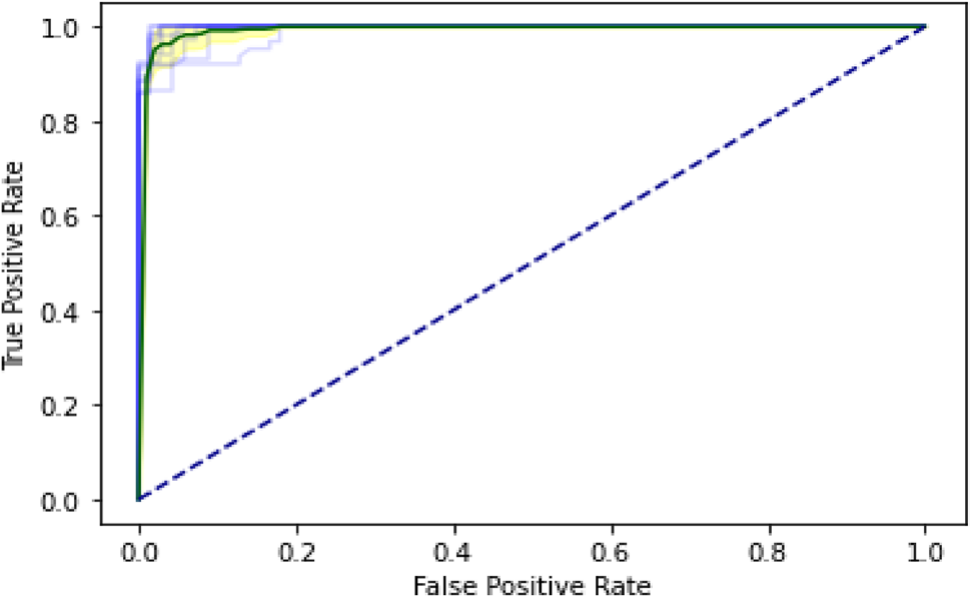
A visual example of receiver-operating (ROC) curve in SCT of BEDLM by using tenfold cross validation test.

The data is evenly subsampled into ten groups using the tenfold cross-validation (FCV) approach. The training set is then partitioned into 10 divisions and treated as a separate validation set, training the model, and then averaging generalization performance over the tenfolds to determine hyper-parameter and architectural decisions^12^. The decision boundary of 10-FCVT is illustrated as following Fig. 13. The ROC curve (receiver operating characteristic curve) of BEDLM in 10-FCVT for all the DL algorithms such as LSTM, GRU, BLSTM is illustrated in the following Fig. 14. The 10-FCVT results are shown in Table 5.

**Table 5:** Results of tenfold cross validation test by using LSTM, GRU, BLSTM and BEDLM.

Deep learning models	Tenfold cross-validation test (10-FCVT)			
	ACC%	SE%	SP%	MCC
GRU	92	91	93	0.84
BLSM	91	93	92	0.84
Proposed BEDLM	93	91	94	0.85
LSTM	93	96	95	0.90

## Discussion

The significance of an automated classification system for skin lesions extends beyond its potential to significantly reduce the workload of dermatologists. By minimizing subjectivity and human error in the classification process, such a system can enhance diagnostic accuracy. The consequences of inaccurate or delayed diagnoses are evident in instances of inappropriate therapy, occasionally necessitating more extensive surgical intervention and prolonged hospital stays. Dermatologists, even with substantial experience, exhibit varying recall rates in skin cancer screening, emphasizing the need for robust diagnostic tools.

In regions where skilled dermatologists are scarce, particularly in developing nations, the proposed automated method can be indispensable. The BEDLM model showcased its effectiveness in automating skin lesion classification, demonstrating the ability to assign class labels to previously unseen lesions. While these findings are promising, further validation and progress hinge on acquiring more clinical data, including factors such as age, gender, race, and family history. Such data are critical before deep learning models can be considered for practical use in clinical settings.

The focus on melanoma detection, a rapidly progressive and extremely dangerous form of skin cancer, underscores the collaborative efforts between medical and computational research. The proposed BEDLM, incorporating approaches such as LSTM, GRU, and bi-directional LSTM, leverages datasets from recent retrospective cohort studies to identify melanoma. This approach aims at enabling early diagnosis, even before visible symptoms manifest.

Utilizing the most recent dataset for normal and mutant gene sequences of cutaneous melanocyte cancer, this study employs three distinct testing methodologies: SCT, 10-FCVT, and IST. The results indicate high accuracy levels, with 94%, 93%, and 97% accuracy in SCT, 10-FCVT, and IST, respectively. Notably, the BLSTM model achieves an impressive 99% accuracy in skin melanoma prediction, showcasing its suitability for high-accuracy applications.

Table 3 provides a comprehensive overview of outcomes for LSTM, GRU, BLSTM, and BEDLM across SCT, IST, and 10-FCVT. The BEDLM, trained on ninefolds and tested on onefold, undergoes repeated iterations, using the complete dataset for both testing and training. The incorporation of randomized data in each iteration enhances learning, with the average accuracy calculated at the conclusion of the process.

Looking ahead, genomic technologies hold promise in predicting subsets of melanoma patients more accurately, both biologically and clinically. These advancements pave the way for personalized medicine, allowing precise identification of molecular alterations in tumor cell populations as the disease progresses.

## Conclusion

Cutaneous melanoma of the skin is a very severe type of cancer with its fast progression. As a result, for early diagnosis, a suggested BEDLM framework as shown in Fig. 7 including these three deep learning algorithms which are GRU illustrated in Fig. 5, LSTM illustrated in Fig. 4, and BLSTM illustrated in Fig. 6 is devised. Normal gene sequences are downloaded from asia.ensembl.org, while mutated gene information is acquired from IntOgen.org with the help of web scraping code. By integrating mutation information into normal gene sequences, mutated sequences are obtained as shown in the Fig. 2 (Data acquisition framework). Proposed study dataset contained 2608 human samples and 6778 mutations in total along with 75 types of different gene as shown in the following Table 2. Multiple feature extraction techniques are used in this study as shown in Fig. 3 (feature extraction) for obtaining features from normal gene and mutated gene sequences and for converting the data for training and testing into numeric format. The BEDLM proposed in this study gives 97% accuracy as shown in Table 3 (Results of SCT, IST and 10-FCVT in LSTM, GRU, BLSTM and BEDLM). To evaluate the performance of the proposed model different testing techniques such as 10-FCVT, SCT and IST are applied. The decision boundary of SCT illustrated in Fig. 9 and its ROC curve illustrated in Fig. 10. The ROC curve of IST illustrated in Fig. 12 and its decision boundary showed in Fig. 11. The decision boundary of 10-FCVT showed in Fig. 13 and its ROC curve illustrated in Fig. 14. Proposed BEDLM shows superior performance in IST. Result comparison of all algorithms such as LSTM, GRU, BLSTM and BEDLM and their accuracy, Sensitivity, Specificity, and Mathew’s correlation coefficient showed in the Table 3. The findings demonstrate that the suggested BEDLM in the proposed study can be used effectively for early diagnosis of cutaneous melanoma of skin.

In the future this technique can be used in identifying other life-threatening diseases and other deep learning models can be used for obtaining more accuracy and efficiency.

## Data Availability

The genomic data utilized in this study is accessible at https://www.intogen.org/search?cancer=MEL and http://asia.ensembl.org/Homo_sapiens/Gene/Sequence?g=ENSG00000141510;r=17:7661779-7687538. The dataset obtained after preprocessing is also available upon request from the first author at alishahsadiq@gmail.com.
